# Report of Case of Malignant Stricture of the Descending Colon

**Published:** 1884-04

**Authors:** J. D. Skeer


### ARTICLE VIII.

REPORT OF CASE OF MALIGNANT STRICTURE OF THE DESCENDING COLON. By DR. J. D. SKEER. Read before the Chicago Pathological Society, and reported by the Secretary.

Mr. F. C., of good family history, and previous good health, suffered from indigestion, constipation or diarrhœa, and general debility, since last September. Nov. 11, stated to Dr. Skeer that he had had no movement for two or three days; mild cathartics were then ordered, but ineffectually.

For the next few days the abdomen remained uniformly distended; dullness in the right iliac region, no tumor or well localized pain, but paroxysmal nervous pain.

Temperature 100°, pulse 76.

The diagnosis was at this time obstruction from hardened fæces at the ileo-cœcal valve, perhaps atony of bowels or spasmodic stricture, possibly organic stricture.

At no time, excpt just before death, did the pulse rise above 80, or the temperature above 101°.

Mild cathartics were again prescribed, followed by copious injections, containing castor oil, etc. These injections were frequently repeated during the progress of the case, but only about two-thirds of the fluid was returned each time. Morphia was given in small doses at bed-time, to relieve pain, relax spasm, and produce sleep.

On the morning of the 14th he passed a number of small scybalæ and a volume of offensive gas. Dr. McClure and Dr. Knox, called in consultation during the next two or three days, both concurred in the diagnosis of obstruction from fæcal accumulation.

On the evening of the 18th, for the first time, the patient vomited fluid stercoraceous matter, and on the morning of the 19th he died. Post-mortem examination six hours after death, was made of the abdominal viscera. The peritoneal sac contained a large amount of yellowish feculent fluid. The undertaker, in embalming the remains, had perforated the bowels with a trocar in a dozen or more places. The small intestines were congested, and the ascending colon very much distended. Two very small perforating ulcers were found in the transverse colon, but no traces of peritoneal inflammation.

In the descending colon, just above the sigmoid flexure, was found an organic structure, almost closing the caliber of the bowels, leaving a passage the size of a goose quill. All fæcal matter which was soluble was thoroughly liquefied, and instead of hardened fæces at point of obstruction, a collection of grape seeds and apple skins, which had acted as a valve, thus preventing the escape of more than two-thirds of the amounts injected.

In regard to the specimen, attention was called to the constricting bands of peritonæum; to the contraction, thickening, and induration of the walls of the bowel; to the circular ulcer above and extending down into the stricture, and to the small caliber of the bowel.

*Note.*—The above specimen having been referred to a committee for microscopical examination, it was reported at the following meeting of the society that the growth was found to consist of round-celled sarcoma, embryonic cells,— rarely found in the alimentary canal, and exceedingly malignant in nature.

J. H. TEBBETTS, Secretary.

